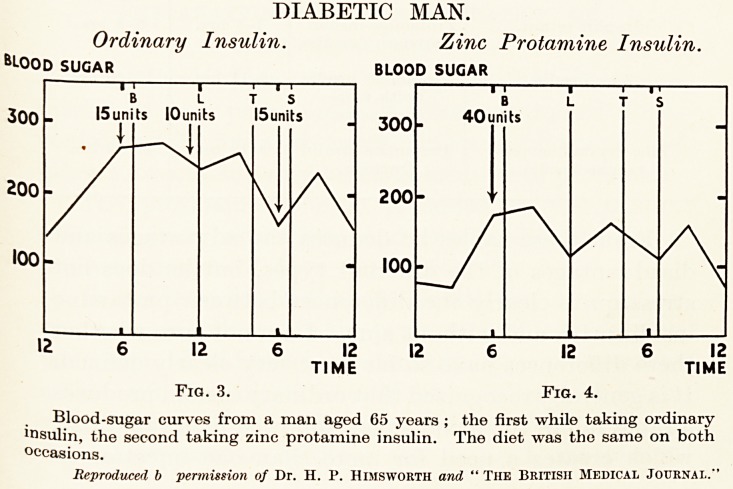# The Use of Protamine Insulin
*A paper read at the Meeting of the British Medical Association (Bath, Bristol and Somerset Branch), on Wednesday, 27th April, 1938.


**Published:** 1938

**Authors:** J. A. Nixon

**Affiliations:** Emeritus Professor of Medicine in the University of Bristol


### THE USE OF PROTAMINE INSULIN.*

BY

J. A. Nixon, C.M.G., M.D., F.R.C.P.,

*Emeritus Professor of Medicine in the University of Bristol.*

---

The recent introduction of delayed action insulin has rendered the treatment of diabetes a good deal more tolerable to the patient without greatly increasing the doctor's difficulties. The older form of insulin was freely soluble and hence was rapidly absorbed. Its effect was rapidly felt and almost as rapidly exhausted. Hence there were frequent hypoglycæmic reactions which alarmed the patients and sometimes led to their refusing to continue a treatment which appeared to be and was in fact dangerous.

In order to avoid sudden falls in blood-sugar various attempts have been made to prolong the action of insulin, and to imitate the natural mechanism of regulation, which always amounts to minute doses being added to the blood in response to calls which we cannot recognize or imitate.

From a study of normal blood-sugar curves it is established that there is a uniform rise during the night, ending with a sharp, steep rise towards morning. During the day numerous exogenous influences lead to irregular rises and falls. The early morning rise which is observed normally becomes much more pronounced during treatment with insulin.

* A paper read at the Meeting of the British Medical Association (Bath, Bristol and Somerset Branch), on Wednesday, 27th April, 1938.

At the beginning of 1936, Hagedorn of Copenhagen introduced a combination of insulin with protamine which was proved to have a much slower action than ordinary insulin. This combination he called protamine insulinate. The compound is made with protamine prepared from the sperm of the rainbow trout (*salmo irideus*).

A still more prolonged effect is obtained by the addition of zinc to protamine insulin. By both these methods an insoluble compound is formed, the insolubility of which is greatest at the reaction of normal plasma pH. 7·3. At this reaction it is injected as a suspension—the deposit in the subcutaneous tissues consists of solid particles, and a fluid of constant insulin concentration is formed from which absorption slowly takes place. Thus its effect is more uniform and of longer duration than ordinary insulin, as may be seen from the following blood-sugar curves :—

FIG. 1.—Shows the blood-sugar curves in a normal man, fasting, after the injection of 30 units of ordinary and of protamine insulin. The dotted line shows the curve after ordinary insulin, the solid line that which follows protamine insulin. It will be seen that after ordinary insulin the blood-sugar is returning to the normal level (even though no food is taken) after the second hour.

After protamine insulin the fall in blood-sugar continues until at the sixth hour it has reached the dangerously low level of 40 mgm. per 100 c.c.

FIG. 2.—Shows the blood-sugar curves in a normal man who takes his ordinary meals, breakfast, lunch, tea and supper, after the injection of 20 units of ordinary insulin and the injection of protamine insulin. The dotted and solid lines have the same meaning as in Fig. 1. It is evident that the effect of protamine insulin is more protracted, and the blood-sugar remains low in spite of breakfast and lunch, whilst it begins to rise just before tea. After ordinary insulin the blood-sugar begins to rise even before lunch.

### NORMAL MAN.

*Fasting.*

FIG. 1.

*Meals.*

FIG. 2.

Comparison between ordinary insulin and protamine (retard) insulin in a normal man. In the fasting man the curves show rapid recovery from effect of ordinary insulin, but protamine (retard) insulin still acting six hours after the injection and producing hypoglycæmic attack. On a fixed diet the curves show the effect of ordinary insulin "buffered" by lunch, but protamine insulin continues to act strongly until its action is "buffered" by tea.

*Reproduced by permission of* DR. IZOD BENNETT *and* "THE LANCET."

### DIABETIC MAN.

*Ordinary Insulin.*

FIG. 3.

*Zinc Protamine Insulin.*

FIG. 4.

Blood-sugar curves from a man aged 65 years; the first while taking ordinary insulin, the second taking zinc protamine insulin. The diet was the same on both occasions.

*Reproduced b permission of* DR. H. P. HIMSWORTH *and* "THE BRITISH MEDICAL JOURNAL."

FIGS. 3 and 4 refer to the effects on a diabetic subject, and demonstrate the contrast between the effects of ordinary and zinc-protamine insulin.

Three injections of ordinary insulin are given as against one of zinc-protamine insulin. The total number of units (45) is the same.

The points to notice are that in Fig. 4 after zinc-protamine insulin the general level of the blood-sugar is much lower, and in particular that the level remains low during the greater part of the night and rises steeply just before breakfast.

### THE DIFFERENCE IN ACTION BETWEEN PROTAMINE INSULIN AND ZINC-PROTAMINE INSULIN.

Dr. Izod Bennett has published a table which sets out the varieties and times of action of the different types of insulin.

TABLE I.

*Types of Insulin.*

| Type of Insulin. | Synonyms. | Commencement of Action. | Maximum Effect. |
|---|---|---|---|
| Ordinary insulin. | — | 20–40 min. | 3 hrs. |
| Insulin retard. | Protamine insulin ; Hagedorn's insulin. | 1–3 hrs. | 6–8 hrs. |
| Zinc insulin. | Protamine insulin (with zinc) suspension. | 9–11 hrs. | 15–20 hrs. |
| Zinc crystalline protamine insulin. | Protamine-insulin Organon. | 8–12 hrs. | 12–20 hrs. |

From these tables he deduces the advantages and disadvantages of the different types, but he does not stress quite clearly the differences between protamine insulin with and without zinc. I am not sure whether these differences have so far been very clearly defined. It is generally recognized that ordinary insulin produces a rapid fall in the blood-sugar, followed by a rapid rise, which creates a need for more than one injection in twenty-four hours in most cases of diabetes. When Hagedorn's protamine insulin is used the fall in blood-sugar begins soon after the first meal following the injection, reaches its maximum in six or eight hours (that is to say, before night if given in the morning), and its effect is completely exhausted before the dose is given next day.

With zinc-protamine insulin the fall in blood-sugar does not begin until nine to eleven hours after the injection, the maximum effect is not reached until fifteen or twenty-four hours after injection, and the effect is not exhausted when the time for the next dose comes round.

The advantage lies with the non-zinc compound, because the end point of its action is reached within twenty-four hours.

My general impression is that at present protamine insulin without zinc is to be preferred to zinc-protamine insulin.

### PREPARATION AND ADMINISTRATION OF PROTAMINE INSULINS.

Hagedorn's plan was to supply the protamine insulin in two phials, one of which contained 4 c.c. containing 50 units of water-soluble insulin, and the other 1 c.c. of a suitably buffered solution of protamine.

The protamine solution is added to the insulin solution just before use, and remains stable for a limited number of days (10 to 14).

It was later found that the addition of zinc to the protamine insulinate, in the proportion of 0·2 mg. zinc per 100 units of insulin, rendered the precipitate more stable, and the injection could be supplied in a single phial. There is a disadvantage in each form. If the 2-phial preparation without zinc is used, the insulin remains potent for at least eighteen months, provided the protamine solution has not been added. After its addition the mixture must be used within a few days. When the zinc-protamine insulin in a single phial is used the suspension only remains potent for six months. This may be a matter of indifference to an individual patient, but it necessitates hospitals and pharmacists keeping only small stocks. In either case the suspension must be shaken up before injection.

At first Hagedorn and his co-workers recommended an injection of protamine insulin at night and one of ordinary insulin in the morning. The use of two injections of protamine insulin daily is certainly inadvisable, because the full effect of the first injection is not exhausted for about twenty-four hours.

The general opinion of those who have had most experience is that the morning before breakfast is the best time for administering protamine insulin. If a single dose is inadequate, a second dose of ordinary insulin can be given in the evening. If the dose is given in the morning there is no reason to fear a hypoglycæmic reaction, because the immediate effect of protamine insulin is small, and before its full sugar-reducing effect is exerted the patient will have had breakfast and the mid-day meal.

In patients who require exceptionally large doses a difficulty arises from the bulk of the injection. At present 40 units per 1 c.c. is the limit. There is some doubt whether a more concentrated solution, say of 80 units per 1 c.c., is absorbed completely.

A single injection of more than 1 c.c. is usually uncomfortable, though in my experience the local reaction is greater with ordinary than with protamine insulin. I have not found patients object to 1·5 c.c., *i.e.* 60 units.

Wilder has pointed out that there is an advantage in giving all the insulin for the day in a single injection before breakfast, because then the qualitative test of the early morning urine for sugar becomes an accurate guide to dosage.

If the morning urine contains sugar the dose of insulin is too small and may be increased with safety. If there is no sugar the dose is adequate, and a hypoglycæmic reaction is an indication for a smaller dose.

Our aim therefore is to secure sugar-free urine in the morning without producing any hypoglycæmic reactions, and this is easier to do with delayed action protamine insulin than with ordinary insulin, which produces a much more rapid fall in blood-sugar.

As regards the initial dosage—if the patient is already taking ordinary insulin one should substitute the same number of units of protamine insulin and give it in a single morning injection. If necessary a supplementary evening dose of about 60 per cent. of the previous dose of ordinary insulin may be given.

Alcohol in the syringe must be avoided.

### MODE OF ONSET OF HYPOGLYCÆMIA.

In any of the forms of delayed action insulin the mode of onset of hypoglycæmia needs to be specially observed. Whereas with ordinary insulin the fall in blood-sugar is abrupt and usually attended by premonitory symptoms which the patient may recognize, the response to treatment with glucose or adrenalin is generally prompt and, even if untreated, the hypoglycæmia is often only transient, so that the patient may recover without any treatment. On the other hand, with delayed action insulin the fall in blood-sugar may be so gradual that no premonitory symptoms are experienced. Moreover, the fall is so slow that a much lower level of blood-sugar has to be reached before any hypoglycæmic symptoms present themselves: the subjective warning symptoms of faintness, perspiration, trembling and emptiness are frequently absent and the patient becomes deeply comatose without other warning.

Since the effect of these types of insulin is so slow the response to treatment can be expected to be slow also. The rapid recovery after adrenalin or glucose does not occur, and when apparent recovery has taken place the effect of the delayed action insulin may outlast the effects of the glucose administered so that repeated administration of glucose is essential. In some reported instances hypoglycæmic symptoms have been delayed until the blood-sugar had reached 40 mgm. per 100 c.c.

It will readily be appreciated that to restore a patient from this degree of hypoglycæmia with insulin-action still unexhausted is much more problematical than in a case where hypoglycæmia manifests itself with the blood-sugar in the neighbourhood of 60 mgm. and the insulin-action known to be short-lasting.

Theoretically we might assume that the most rapidly acting form of insulin should be employed to combat coma. The *Lancet*, in an editorial (6th March, 1937), said that most authorities agree that ordinary insulin should always be preferred for this most dangerous complication.

Experience at the Mayo Clinic, and that of Rabinowitch and his colleagues, suggest that protamine insulin may be successfully employed in cases of severe acidosis. But the practice at the Mayo Clinic is to give from 50 to 100 units of protamine insulin without delay, and to follow this up with multiple doses of ordinary insulin as may be indicated by examinations of the blood-sugar and the urine.

### DIETS WITH PROTAMINE INSULIN.

All observers are agreed that the diet need not be so strictly regulated with protamine insulin. The patient may be allowed a good deal more latitude, and may to some extent be guided by his own appetite as regards the amount eaten at a particular meal. For some years past I have allowed diabetic patients to determine the amount they eat. I let them select their own diets within reason, and ask them to keep to approximately the same amounts for each day. The size of each meal may vary considerably so long as the total intake for the day does not vary materially.

Some recent work by McCance and Widdowson at

TABLE II.

*Daily Food Intake of Sixty-three Men of the Middle Class. Summary of Results.*

| | Average. | Maximum. | Minimum. | Standard Deviation. |
|---|---|---|---|---|
| Total calories per day .. | 3,067 | 4,955 | 1,772 | 714 |
| Calories per kg. body weight (normal weight for height and age) .. .. .. .. | 43·8 | 74·6 | 25·2 | 11·3 |
| Total protein (g. per day) .. | 97·6 | 167 | 53 | 23·8 |
| Animal protein (g. per day) .. | 66·6 | 121 | 30 | 19·2 |
| Total fat (g. per day).. .. | 129·4 | 215 | 69 | 35·0 |
| Total carbohydrate (g. per day) | 348 | 589 | 171 | 86·0 |
| % of calories from protein .. | 13·1 | 19·3 | 8·3 | 1·9 |
| % of calories from fat .. | 39·1 | 50·8 | 29·0 | 4·9 |
| % of calories from carbohydrate | 46·7 | 59·8 | 33·5 | 5·4 |
| Calcium (g. per day) .. .. | 0·87 | 1·96 | 0·36 | 0·36 |
| Total phosphorus (g. per day) | 1·61 | 2·79 | 0·87 | 0·44 |
| " Available " (non-phytin) phosphorus (g. per day) .. | 1·57 | 2·73 | 0·86 | 0·42 |
| Total iron (mg. per day) .. | 16·8 | 28·5 | 7·8 | 4·64 |
| " Available " (inorganic) iron (mg. per day) .. .. | 10·8 | 18·7 | 5·3 | 2·80 |
| Intake of milk (pints per day).. | 0·58 | 1·46 | 0 | 0·32 |
| Intake of meat (oz. per day) .. | 5·2 | 11·1 | 1·4 | 2·0 |
| Intake of bread (oz. per day) .. | 7·6 | 16·2 | 0·6 | 3·4 |
| Calories per penny .. .. | 117 | 165 | 53 | 23·6 |

N.B.—The sum of the percentages of calories derived from protein, fat and carbohydrate is less than 100 because a small percentage of the calories is derived from alcohol.

From " A Study of English Diets by the Individual Method," Part I, Men, by E. M. Widdowson, *Journal of Hygiene*, vol. xxxvi., 1936, p. 274.

King's College Hospital has thrown a great deal of light on the individual variations in the selection of adequate diets. They have studied the daily food intake of sixty-three men and sixty-three women of middle class, whose circumstances were such that financial considerations did not necessitate any undue economies. The resulting maxima and minima are astonishing. Each individual considered himself or herself adequately nourished for carrying on his or her daily work. Their weights remained steady. Those who ate largely did not seem overfed, and those who ate sparingly did not appear undernourished.

TABLE III.

*Daily Food Intake of Sixty-three Women of the Middle Class. Summary of Results.*

| | Average. | Maximum. | Minimum. | Standard Deviation. | Average expressed as a percentage of the mean value for men. |
|---|---|---|---|---|---|
| Total calories per day .. | 2,187 | 3,110 | 1,453 | 388 | 71 |
| Calories per kg. body weight (normal weight for height and age) .. .. .. | 35·5 | 55·6 | 26·6 | 7·6 | 81 |
| Total protein (g. per day) .. | 67·3 | 90·0 | 28·0 | 12·4 | 69 |
| Animal protein (g. per day) | 46·0 | 64·0 | 9·0 | 11·3 | 69 |
| Total fat (g. per day) .. | 100·5 | 151 | 63 | 20·6 | 77 |
| Total carbohydrate (g. per day) .. .. .. | 233 | 384 | 113 | 58·5 | 67 |
| % of calories from protein .. | 12·8 | 17·3 | 6·9 | 2·2 | — |
| % of calories from fat .. | 42·7 | 54·9 | 32·6 | 4·7 | — |
| % of calories from carbohydrate .. .. .. | 43·6 | 56·5 | 16·7 | 6·0 | — |
| Total calcium (g. per day) .. | 0·63 | 1·16 | 0·23 | 0·16 | 72 |
| Total phosphorus (g. per day) | 1·13 | 1·72 | 0·48 | 0·25 | 70 |
| " Available " (non-phytin) phosphorus (g. per day) .. | 1·09 | 1·62 | 0·45 | 0·22 | 69 |
| Total iron (mg. per day) .. | 11·4 | 17·3 | 5·5 | 2·50 | 68 |
| " Available " (inorganic) iron (mg. per day) .. .. | 7·9 | 12·4 | 5·0 | 1·64 | 73 |
| Intake of milk (pints per day) | 0·45 | 0·86 | 0 | 0·17 | 78 |
| Intake of meat (oz. per day) | 3·0 | 6·0 | 0·1 | 1·2 | 60 |
| Intake of bread (oz. per day) | 4·5 | 8·1 | 1·2 | 1·6 | 56 |
| Calories per penny .. .. | 109 | 166 | 29 | 21·4 | — |

From " A Study of English Diets by the Individual Method," Part II, Women, by E. M. Widdowson and R. A. McCance, *Journal of Hygiene*, vol. xxxvi., 1936, p. 299.

My own experience is that diabetic patients, when allowed to select their own diets—and sugar or jam are deliberately excluded—rarely take as much as 3,000 calories daily for men and 2,000 calories daily for women. Speaking generally, a diet moderately low in carbohydrate is more easily controlled by a single dose of insulin than a very generous carbohydrate diet. This is just as might be expected.

## Figures and Tables

**Fig. 1. Fig. 2. f1:**
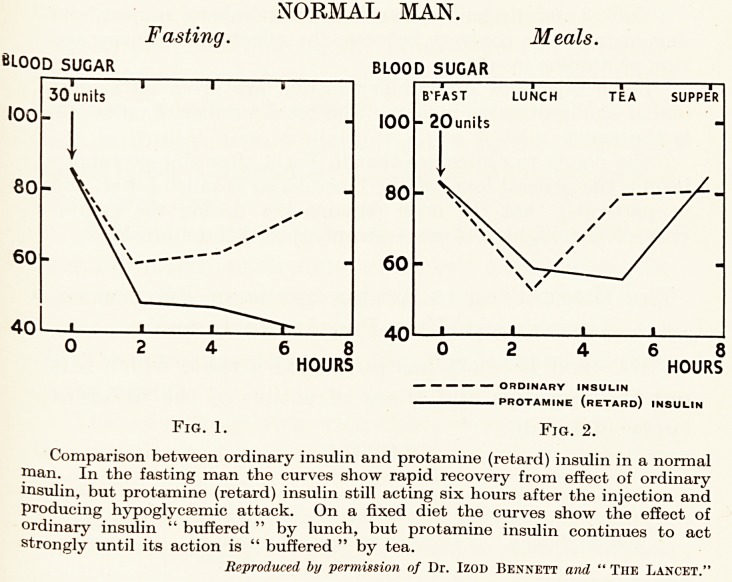


**Fig. 3. Fig. 4. f2:**